# An Autogenously Regulated Expression System for Gene Therapeutic Ocular Applications

**DOI:** 10.1038/srep17105

**Published:** 2015-11-24

**Authors:** Matthew A. Sochor, Vidyullatha Vasireddy, Theodore G. Drivas, Adam Wojno, Thu Doung, Ivan Shpylchak, Jeannette Bennicelli, Daniel Chung, Jean Bennett, Mitchell Lewis

**Affiliations:** 1 Center for Advanced Retinal and Ocular Therapeutics, F. M. Kirby Center for Molecular Ophthalmology, Philadelphia, PA 19104, USA; 2 Department of Biochemistry and Biophysics Perelman School of Medicine, University of Pennsylvania, Philadelphia, PA 19104, USA

## Abstract

The future of treating inherited and acquired genetic diseases will be defined by our ability to introduce transgenes into cells and restore normal physiology. Here we describe an autogenous transgene regulatory system (ARES), based on the bacterial lac repressor, and demonstrate its utility for controlling the expression of a transgene in bacteria, eukaryotic cells, and in the retina of mice. This ARES system is inducible by the small non-pharmacologic molecule, Isopropyl β-D-1-thiogalactopyranoside (IPTG) that has no off-target effects in mammals. Following subretinal injection of an adeno-associated virus (AAV) vector encoding ARES, luciferase expression can be reversibly controlled in the murine retina by oral delivery of IPTG over three induction-repression cycles. The ability to induce transgene expression repeatedly via administration of an oral inducer *in vivo*, suggests that this type of regulatory system holds great promise for applications in human gene therapy.

The field of gene therapy has seen many significant advances over the past decade^1^,^2^,^3^. Viral vectors are available that can effectively express heterologous genes *in vivo* and provide long-term gene expression to target tissues with minimal toxicity and immune response. One of the most successful applications of this technology has been the restoration of vision for individuals with the retinal degenerative disorder described as Leber’s congenital amaurosis (LCA)^4^,^5^. Currently there are approximately 1800 ongoing human gene therapy clinical trials that use similar gene replacement therapies; however, for many pathological conditions gene augmentation therapies need to be regulated^1^. Indeed, gene regulation remains one of the most important and unresolved obstacles for safe and effective development of clinical gene therapeutics.

Most transgene regulatory systems are based upon the classical bacterial operons, where a regulating protein is constitutively produced by one promoter to modulate transcription of a second promoter expressing a functional gene^6^. A variety of such inducible regulatory systems have been developed utilizing a number of different regulatory proteins. These regulatory proteins are allosterically controlled by effector molecules such as the antibiotic tetracycline (Tet), steroid hormones (ecdysone), anti-steroid hormone analogs (mifepristone and tamoxifen), and immunosuppressant (rapamycin)^7^. Regardless of how the regulator is controlled, the fundamental problems that have plagued all of these regulatory systems is that effector molecules can produce unwanted side effects, the regulatory circuitry exhibits a high basal level of gene expression with only a modest dynamic range, and many of these systems are too large to easily fit within the packaging constraints of a single viral vector such as a recombinant adeno-associated virus (rAAV). Additionally, the level of regulator protein produced in these systems is constant and dependent on a number of extrinsic variables, such that the system will behave differently in different environments. The resulting switch must be empirically tuned for each particular application to ensure sufficient dynamic range and to minimize leakiness of transgene expression in the uninduced state.

An alternate approach for regulating gene expression, used by many biosynthetic operons, is autogeny^8^,^9^. Autogenous systems rely on a single regulated promoter that controls the expression of both the regulatory protein as well as the functional gene. The fundamental advantage of autogeny is that it eliminates the need to balance promoter productivity. In theory, autogenous expression of the regulator protein ensures a steady state level of a transcriptional regulator, regardless of the model system, cell type, copy number, or functional gene. Moreover, autogenous regulation has been shown to exhibit a linearized effector response (rheostatic)^10^, have a quicker response upon effector dosage (sensitivity)^11^, and is inherently less noisy than classically regulated gene circuits (stable)^12^. An additional practical advantage of an autogenously regulated system for gene replacement therapy is that using a single promoter significantly increases the effective cargo capacity of the viral delivery vector. For some vectors, such as AAV, the size of the vector poses a real physical limitation. The utility of autogeny has been recently demonstrated using a tetracycline regulated promoter^13^, however, there have not been any direct comparisons of autogenously and classically regulated expression systems. Here we compare classical and autogenous regulation in a variety of model systems and explore the utility of autogenous regulation for gene therapy.

To evaluate the regulatory properties of an autogenously regulated expression system, we first compared the induction profiles of constitutively and autogenously expressed reporter protein in bacteria, with both systems utilizing the *lacI* repressor protein of the lac operon as the transcriptional regulator. The classically regulated expression system (CRES) was constructed using a weak constitutive bacterial promoter (p*LacI*) to drive the expression of the *lacI* repressor protein fused to mCherry. This repressor regulates a stronger *inducible* promoter (p*Lac*) expressing yellow fluorescent protein (YFP) (Fig. 1A). The autogenously regulated expression system (ARES) relied on the same pLac inducible promoter to drive expression of a polycistronic message containing both YFP and the *lacI* protein fused to mCherry (Fig. 1B). Induction profiles were measured as a function of inducer (isopropyl thiogalactoside, IPTG), and both regulatory systems demonstrated inducible YFP expression, as expected, producing low levels of YFP in the absence of inducer and showing nearly full induction at low millimolar IPTG concentrations (Fig. 1C). In ARES, mCherry fluorescence, indicative of levels of mCherry-tagged *lacI* repressor protein, was also found to be significantly inducible with increasing doses of IPTG, confirming that in autogenously regulated systems repressor protein levels are appropriately titrated to the given conditions (Fig. 1D). We did also notice a very small increase in mCherry fluorescence in CRES with increasing inducer concentrations, and suspect that this is simply due to an overlap of the emission spectra of YFP and mCherry rather than a true increase in levels of *lacI*-mCherry fusion protein.

One significant advantage of the ARES system was immediately apparent in our data: whereas ARES automatically titrates repressor protein levels to maintain appropriate levels, CRES cannot fine-tune it’s own expression for different inducer levels or model systems. This was apparent when looking both at repressor levels and at the leakiness of our systems. Levels of repressor in CRES, as indicated by mCherry fluorescence, were too low (nearly 5-fold lower than in ARES at the maximally induced state (Fig. 1D)) to maintain tight control of YFP expression, and thus CRES was characterized by significant leakiness of YFP expression when uninduced (Fig. 1C). On the other hand, the lower levels of repressor protein also afforded CRES a greater induction profile than ARES, with YFP levels nearly double those of the autogenous system in the maximally induced state (Fig. 1C). Thus, ARES was found to be less leaky and more adaptable, but with a smaller fold change in YFP expression than CRES, as would be expected^14^. Given the kinetic advantages of autogeny and the appropriate thermodynamic properties, we next wanted to establish how ARES would function in eukaryotic cells.

To establish how these expression systems behave in eukaryotic cells, we created eukaryotic expression systems that are classically and autogenously regulated and compared their regulatory properties in transfected HEK293T cells. Analogous to the prokaryotic system, a constitutively regulated expression system (CRES) was produced using a bi-directional expression system (Clontech, Mountain View, CA) where a weak minimal cytomegalovirus promoter (P_minCMV2_) drives expression of a eukaryotic codon-optimized lac repressor^15^ and a second, stronger minimal cytomegalovirus promoter (P_minCMV1_), modified by inserting the high affinity symmetric lac operator sequence^16^ between the TATA box and the transcription start site, drives inducible expression of the YFP reporter gene (Fig. 1E). An autogenously regulated expression system (ARES) was built using the same modified inducible CMV promoter (P_minCMV1_) to drive expression of a transcript that codes for both the lac repressor and the YFP reporter, linked via a 2A peptide cleavage sequence^17^ (Fig. 1F). These expression systems were transiently transfected into HEK293T cells and YFP fluorescence was measured as a function of the inducer (Fig. 1G). Upon induction, gene expression increased roughly 2.5-fold (p < 0.001) in both systems, confirming that the lac repressor is functional in eukaryotic systems. The induction profiles were similar to what we had observed in prokaryotes, with ARES exhibiting lower levels of expression when maximally induced and slightly lower fold change than what was observed for CRES, but again with significantly lower leakiness in YFP expression in the uninduced state (Fig. 1G).

The autogenously regulated system was further evaluated by replacing YFP with luciferase as a reporter and by the addition of an ancillary operator sequence within the intron, a modification that previous studies have reported to result in tighter transgene regulation^18^. This new construct was sub-cloned into an AAV production vector^19^, and packaged into AAV8 virions (AAV8.ARES.luciferase) (Fig. 2A). HEK293T cells were transduced with AAV8.ARES.luciferase and induction profiles were measured as a function of inducer. Again we observed a robust dose-response (Fig. 1H), this time with a nearly 4-fold induction of luciferase activity between the off and maximally induced states (p < 0.05), confirming that the ARES, when delivered virally, can also successfully regulate eukaryotic gene expression. Once again, an advantage of the ARES system is that it can be tested after incorporation in an AAV vector. In contrast, we were unable to test the constitutively regulated system in AAV because it is too large to fit within the AAV cargo space.

To examine the utility of this ARES *in vivo*, eight, age-matched, adult CD-1 mice received unilateral subretinal injections with AAV8.ARES.luciferase. These mice were then subjected to cycles of IPTG gavage for three days at doses that we had previously found to result in sufficient tissue concentrations of inducer for ARES induction (Fig. S1), followed by at least 5 days without IPTG gavage. Luciferase levels were determined using an IVIS Series Pre-clinical *in vivo* Imaging System immediately before and immediately after each cycle of IPTG administration over a total of three induction cycles during a period of 33 days (Fig. 2B).

Injected retinas displayed substantial luminescence localized to the injected eye, with the intensity of the signal being significantly increased following IPTG dosage (Fig. 2B,D); the un-injected eyes never showed luminescence (Fig. 2C). For each induction cycle, we observed a significant increase in integrated retinal luciferase signal over baseline luminescence when induced (p < 0.01, p < 0.001, and p < 0.001, respectively) and a return to baseline upon withdrawal of IPTG (p < 0.05 and p < 0.001, respectively) (Fig. 2D). The first induction was the most robust, with an average 8-fold increase in signal, whereas the second and third inductions showed a 2-3-fold increase in signal, similar to results we had observed in cultured cells (Fig. 2E). Histological evaluation of retinal sections from both injected and un-injected eyes did not reveal any abnormalities or immune infiltrates, suggesting that the autogenous regulation is effective and well tolerated in the murine retina (Fig. 2F).

Here we have established that an autogenously regulated expression system exhibits similar steady state induction profiles compared to the classical operon model system. Although both regulatory systems successfully control transgene expression in a variety of cell types both *in vitro* and *in vivo*, the autogenous system may be more useful for gene therapy. While a two-promoter system has the desired effect of being able to regulate the transgene and the repressor independently, if the promoters are not properly balanced the switch is ineffective; too much repressor prevents transgene production while transgene production is effectively constitutive if there is too little repressor. The simple architecture of the autogenous system ensures a proper balance of the regulator to minimize leakiness and maximize dynamic range regardless of the system being tested and without the need to empirically balance promoters. On a more practical note, the compact nature of the autogenous system lends itself more readily to packaging within viral vectors for gene therapeutic applications.

A variety of inducible systems that rely on small bioactive effector molecules have been developed for regulating transgene expression. A lac repressor-based system, however, such as the one described here, has a variety of features that are potentially superior for regulating transgene production. The binding affinity of this repressor is modulated with the addition of a metabolite that is non-toxic with no known off-target effects. The allosteric properties of the lac repressor can be tuned to both decrease basal expression and to increase the induction ratio^20^. Such allosteric modifications, along with optimization of promoter/terminator machinery and operator DNA number and placement could improve the performance of the ARES described here while still maintaining its compact size. Finally, lac repressor binds cooperatively to appropriately spaced operators, which in bacteria decrease the leakiness and greatly improve its regulatory properties – this strategy could similarly be applied to the ARES we describe here to affect improved dynamic range and decreased leakiness.

Building a regulatory circuit specifically for gene therapy requires optimizing several variables: The regulatory system needs have a large dynamic range, respond quickly to changes in effector concentration, and must be small, providing sufficient space for the therapeutic gene, given the limited capacity of a viral capsid. An autogenously regulated system satisfies these objectives and, as we have shown here, is comparable in kinetics and dynamic range to traditional constitutively expressed transgene regulatory systems without the need for promoter balancing or empiric tuning for different applications. These data establish the proof-of-concept of using autogeny and the lac repressor to control transgenes in AAV-mediated gene therapeutic applications.

## Methods

### Bacterial Strains and Media

The strain EPB229 (E. coli F- λ- ilvG- rfb-50 rph-1 Δ(lacI-lacA)::frt) was used for prokaryotic YFP regulation assays. This strain was made by the laboratory of Dr. Mark Goulian and derived from MG1655 (E. coli F- λ- ilvG- rfb-50 rph-1). This strain is the “wild-type” K-12 strain and has a total deletion of the lac operon allowing for a clean background within which to study our lac genetic regulatory systems. Liquid media for YFP regulation assays cells used LB media supplemented with appropriate concentrations of antibiotics and the inducer isopropyl thiogalactoside (IPTG).

### Plasmid Construction

**Figure 2:**
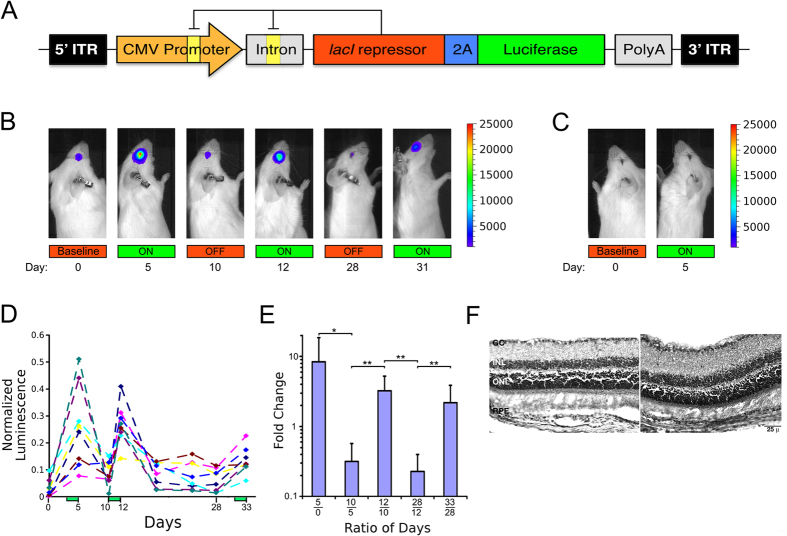
The autogenous regulatory system is functional in mouse retina *in vivo*. (**A**) Map of the autogenously regulated expression system (ARES) within an AAV production vector (AAV8.ARES.Luciferase). A CMV promoter controls the expression of both the *lacI* repressor and Luciferase, linked via a 2A peptide cleavage sequence. Orange boxes indicate lac operator sites. Intronic, polyadenylation, and AAV ITR sequences are indicated. (**B**) Live imaging of luciferase activity over a 33-day period in a representative animal subretinally injected with AAV8.ARES.luciferase in the right eye. (**C**) Live imaging of luciferase activity in the left, un-injected eye of the same animal as in panel (**A**). (**D**) Normalized integrated luminescence of the injected (right) eye were calculated by dividing the observed luminescence by the sum of luminescent measurements made in both the on and off states for each animal. Induction of luciferase in AAV injected eye increases significantly after administration of IPTG (P < 0.01). Green bars represent days of IPTG gavage. (**E**) The fold change was determined by evaluating the normalized integrated luminescence on day n relative to day m, where n and m are labeled on the x-axis as 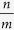 to illustrate dynamic regulation. A fold change >1 indicates induction of luciferase expression while fold change <1 indicates repression of luciferase expression. *p < 0.05, **p < 0.01, n = 8. (**F**) Histological sections of injected retinas from two representative animals stained with hematoxylin and eosin. (RPE, retinal pigmented epithelium, ONL, outer nuclear layer, INL, inner nuclear layer, GC, ganglion cell layer).

The prokaryotic constitutive lac repressor and YFP reporter plasmids were made as previously described^21^,^22^,^23^. Prokaryotic autogenously regulated reporter plasmid was made with the O1 operator sequence (5′-AA TT GTG AGC GAT AAC AA TT-3′) followed by YFP, a 15 base pair spacer (5′-AAT TCA GGG TGG TGA-3′) followed by the lac repressor with a C-terminal mCherry tag that was added to the gene after an 11 bp linker to create the Lac-mCherry fusion protein. The classical eukaryotic gene regulatory plasmid was constructed using the commercially available pBI-CMV1 plasmid (Clontech, Mountain View, CA) by subcloning a eukaryotic codon-optimized lac repressor gene linked at it’s 3′ end to an in-frame mCherry gene followed by a nuclear localization sequence (NLS) into the MCS immediately following the P_minCMV2_ promoter. The gene encoding YFP was subcloned into the MCS immediately following the P_minCMV1_ promoter, which we had modified by inserting the high affinity symmetric lac operator sequence^16^ between the TATA box and the transcription start site. The autogenously regulated eukaryotic gene regulatory plasmid was constructed using the same pBI-CMV1 plasmid. The modified (as above) inducible P_minCMV1_ promoter was used to drive expression of a transcript coding for both the Eukaryotic codon-optimized lac repressor linked to mCherry and an NLS (as above) in addition to the YFP reporter, with the two genes linked via a 2A peptide cleavage sequence^17^. To generate the eukaryotic ARES system expressing luciferase, the entire construct as depicted in Fig. 2A was synthesized by DNA2.0 and subcloned via restriction enzyme digestion and ligation into a previously described AAV production plasmid^19^.

### Prokaryotic YFP and mCherry E. coli regulation assay

We transformed the autogenously regulated reporter plasmid into EPB229 cells (F-Δ(lacI-lacA)::frt). These cells were derived from the MG1655 “wild type” line. Colonies were picked in triplicate into LB media with AMP and CAM and grown overnight at 37 °C with shaking. 50 μL of the overnight culture was used to inoculate 0.5 mL fresh LB media supplemented with varying amounts of IPTG. We measured optical density at 600 nm (OD600), in addition to YFP and mCherry fluorescence for all wells after 24 hours of growth using a TECAN M1000 plate reader in 96 well optical bottom plates (Corning, Corning, NY). All data points collected were normalized to OD600 to account for differences in culture density.

### Eukaryotic Cell Culture, Transfection, Transduction, and Induction

HEK293T cells were grown in Dulbecco’s modified Eagle’s medium (DMEM) supplemented with 10% fetal bovine serum (FBS). All cells were grown in humidified 5% CO_2_ incubators at 37 °C. All transfections were carried out with Fugene 6 reagent following the manufacturer’s protocol. Cells were infected with AAV8.ARES.Luciferase at a concentration of 1 × 10^6^ vg/cell. Cell media was supplemented with various concentrations of IPTG and cells were harvested 48 hours post-transduction/transfection and processed for luminescence/fluorescence analysis.

### Mammalian cell luminescence and fluorescence assays

For luminescence assays, cells were washed with DPBS, lysed with Reporter Lysis Buffer (Promega, Madison,WI), and 10 μL of whole cell lysate was added to 80 μL of luciferase assay buffer (Promega, Madison,WI) containing luciferin within a well of a 96 well optical bottom plate (Corning, Corning, NY). Plates were immediately loaded into a Tecan M1000 instrument and luciferase signal from each well was quantified. For fluorescence assays, cells were washed with DPBS and dislodged from the culture vessel by vigorous pipetting. Cells were resuspended in 50 μL of DPBS and added to individual wells of a 96 well optical bottom plate (Corning, Corning, NY) and YFP fluorescence (excite: 510 nm emit: 535 nm) for all wells was determined using an EnVision® Xcite Multilabel Reader (PerkinElmer).

### Mouse husbandry

Animals were housed in a 12 hour light:dark cycle facility. Animal care was in compliance with the Association for Research in Vision and Ophthalmology statement for the Use of Animals in Ophthalmic and Vision Research, and all procedures were approved performed with approval by the local Institutional Animal Care and Use Committee and were in compliance with federal guidelines.

### Subretinal injections

Subretinal injections of AAV vectors were performed in 6 months old CD1 mice at a dose of 1 × 10^10^ Vg. All surgeries were performed under inhaled anesthesia, and all efforts were made to minimize suffering. Contralateral eyes were used as uninjected controls. Injections were performed as described previously^5^.

### Administration of IPTG

Induction of luciferase expression was accomplished by oral gavage of 2 doses of IPTG twice a day over a 3 day period, with each dose consisting of 25 μl of 1 M IPTG/10 g body weight.

### Bioavailability of IPTG

Three CD-1 adult mice were gavaged with 25 μL of 1 M IPTG per 10 g of body weight twice a day for 3 days. Animals were then sacrificed and major organs were harvested, mechanically homogenized, and steady state concentrations of IPTG in liver, kidney, skeletal muscle, and retina were determined using a beta-galactosidase assay^24^.

### *In vivo* animal imaging

All mice were imaged before each cycle of IPTG gavage, immediately after each cycle of IPTG gavage, and again after 5–8 days of IPTG abstinence. The D-luciferin substrate (Goldbio, St Louis, MO) was injected intraperitoneally, at a dose of 15 μg/g of body weight. Animals were then anesthetized using isofluorane and imaging began 10 min after administration of D-luciferin. The mice were then placed in a light-tight chamber, and images were generated using a cryogenically cooled charge-coupling device camera IVIS 100 (Xenogen, Alameda, CA). Grey scale surface images of mice were collected, and the *in vivo* bioluminescence was represented as a pseudocolor images. The visual output represents the number of photons emitted/second/cm^2^ as a false color image, where the maximum is red and the minimum is dark blue.

### Tissue fixation, cryosectioning and histology

At the end of final IPTG administration, animals were imaged and sacrificed. Eyes were collected and fixed in 4% paraformaldehyde. Tissues were then cryoprotected and embedded in optimal cutting temperature media (Fisher Scientific Co., Pittsburgh, PA, USA) and frozen. Cryosections were made using a Leica CM1850 cryostat (Leica Microsystems, Wetzlar, Germany). Sections were then stained with hematoxylin and eosin (H&E).

### Mouse Retinal Data Analysis

Custom image analysis software was written in Matlab (Mathworks) and the files can be found on the Matlab File Exchange File ID: # 48972. All analyses were performed on 5-second exposure raw luciferase images. Background was measured from an image of an un-injected, negative control left eye and the mean and standard deviation was recorded. A background threshold was defined as background mean +6 standard deviations; pixels higher than this are considered to be true luciferase signal from the AAV8.ITRS.luciferase vector. To isolate a single mouse eye, a region of interest is drawn around the entire mouse head and all pixels above the threshold are recorded. Each pixel intensity has the background mean subtracted and then is summed to integrate over the entire eye. Integrated values were used for statistics and are reported in the text and figures.

### Statistics

Statistical analysis of fold changes in bacterial experiments, eukaryotic tissue culture experiments and mouse experiments were carried out using a Student’s T-test using Gnumeric (gnumeric.org).

## Additional Information

**How to cite this article**: Sochor, M. A. *et al.* An Autogenously Regulated Expression System for Gene Therapeutic Ocular Applications. *Sci. Rep.* **5**, 17105; doi: 10.1038/srep17105 (2015).

## Figures and Tables

**Figure 1:**
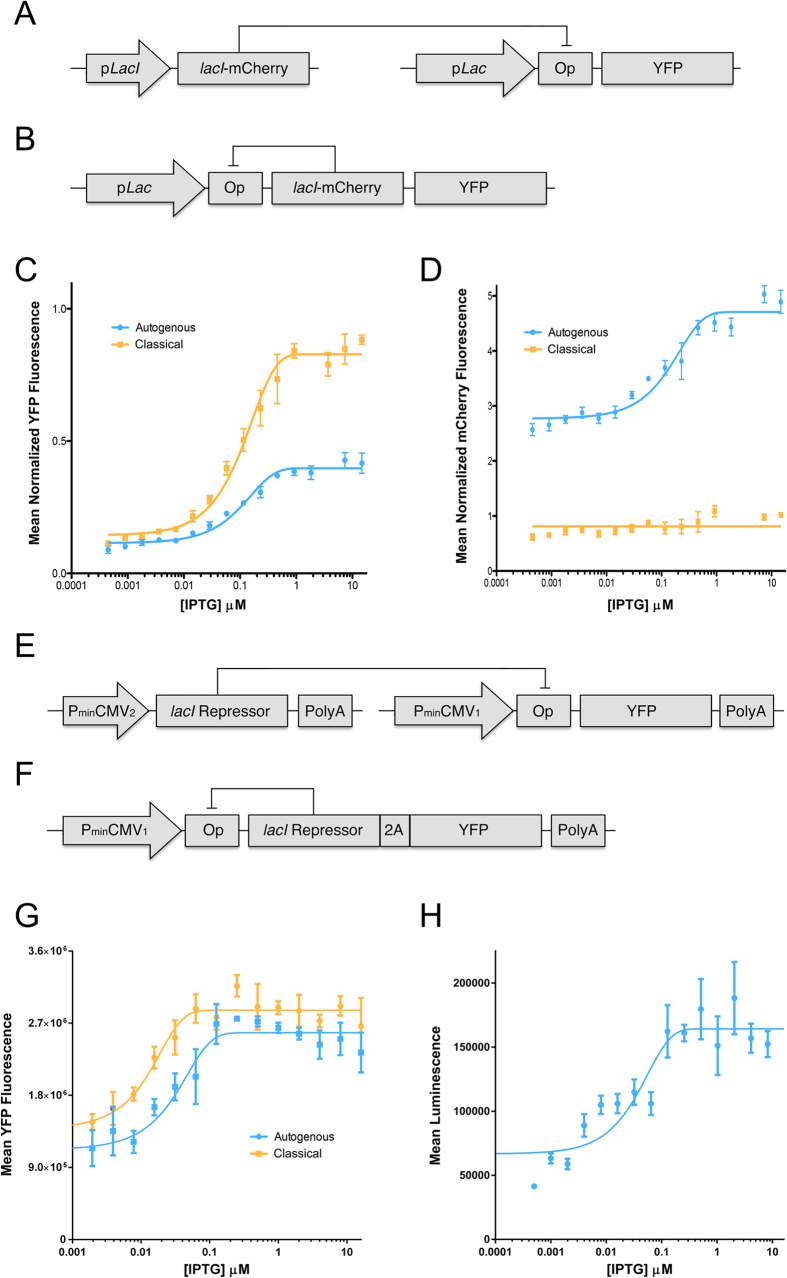
Comparison of constitutively and autogenously regulated transgene systems in *E. coli* and HEK293T cells. (**A, B**) Schematic diagram of the bacterial (**A**) classically regulated expression system (CRES) and (**B**) autogenously regulated expression system (ARES) tested in panels (**C, D**). Promoters, regulator genes, reporter genes, and operator sequence are indicated. (**C**) Mean normalized YFP fluorescence as a function of IPTG concentration for both the CRES and ARES in *E. coli*. Data were normalized to an *E. coli* tranformant expressing YFP under the control of a constitutive promoter. Data points represent mean +/− SEM, n = 5. (**D**) Mean normalized mCherry fluorescence of the *lacI*-mCherry fusion as a function of IPTG concentration for both CRES and ARES in *E. coli.* Data were normalized to an *E. coli* tranformant expressing *lacI*-mCherry under the control of a constitutive promoter. Data points represent mean +/− SEM, n = 5. (**E, F**) Schematic diagram of the eukaryotic (**A**) classically regulated expression system (CRES) and (**B**) autogenously regulated expression system (ARES) tested in panel (**G**). Promoters, regulator genes, reporter genes, polyadenylation sites, 2A cleavage signal, and operator sequence are indicated. (**G**) Mean YFP fluorescence as a function of IPTG concentration for both the CRES and ARES in transfected 293T cells. Data points represent mean +/− SEM, n = 3. (**H**) Mean luminescence as a function of IPTG concentration for the ARES encoding luciferase as a reporter in AAV-transduced 293T cells. Data points represent mean +/− SEM, n = 3.
