# Author Correction: An Autogenously Regulated Expression System for Gene Therapeutic Ocular Applications

**DOI:** 10.1038/s41598-018-35960-w

**Published:** 2018-11-23

**Authors:** Matthew A. Sochor, Vidyullatha Vasireddy, Theodore G. Drivas, Adam Wojno, Thu T. Duong, Ivan Shpylchak, Jeannette Bennicelli, Daniel Chung, Jean Bennett, Mitchell Lewis

**Affiliations:** 1 Center for Advanced Retinal and Ocular Therapeutics, F. M. Kirby Center for Molecular Ophthalmology, Philadelphia, PA 19104 USA; 2 Department of Biochemistry and Biophysics Perelman School of Medicine, University of Pennsylvania, Philadelphia, PA 19104 USA

Correction to: *Scientific Reports* 10.1038/srep17105, published online 24 November 2015

This Article contains an error in the spelling of the author Thu T. Duong, which was incorrectly given as Thu Doung.

